# Increased polyclonal immunoglobulin reactivity toward human and bacterial proteins is associated with clinical protection in human *Plasmodium *infection

**DOI:** 10.1186/1475-2875-4-5

**Published:** 2005-01-20

**Authors:** Constantin Fesel, Luis F Goulart, Adolfo Silva Neto, Alysson Coelho, Cor Jesus F Fontes, Erika M Braga, Nelson M Vaz

**Affiliations:** 1 Instituto Gulbenkian de Ciência, Apartado 14, 2781-901 Oeiras, Portugal; 2 Dept. Bioquimica-Imunologia, ICB, Universidade Federal de Minas Gerais, Belo Horizonte, Brazil; 3 Dept. de Clínica Médica, Universidade Federal de Mato Grosso, Cuiaba, Brazil; 4 Dept. Parasitologia, ICB, Universidade Federal de Minas Gerais, Belo Horizonte, Brazil

## Abstract

**Background:**

Polyclonal B-cell activation is well known to occur in *Plasmodium *infections, but its role in pathogenesis or protection remains unclear. However, protective properties of natural antibodies have previously been demonstrated in other contexts.

**Methods:**

Sera from asymptomatic and symptomatic *Plasmodium*-infected subjects locally detected in a survey study in the Brazilian Amazon, and from unexposed and exposed but presently uninfected control subjects, were assayed by a standardized quantitative immunoblot method allowing simultaneous detection of IgG or IgM reactivity to a large number of parasite-unrelated proteins.

**Results:**

In subjects free of coinfection with hepatitis B virus, IgG reactivity to human brain antigens and *Escherichia coli *proteins was strikingly enhanced in asymptomatic *Plasmodium*-infected individuals when compared to such with clinical malaria symptoms, or to uninfected control subjects. This difference was most characteristic for limited exposure times (less than ten years locally, or 20 years in endemic areas). It was more significant than a similar trend found for IgG to *Plasmodium falciparum *antigens, and unrelated to parasitaemia levels. Asymptomatic subjects with comparatively short exposure characteristically showed relatively elevated IgG versus IgM reactivity. Polyclonal IgG reactivity appears triggered by previous *P. falciparum *but not *Plasmodium vivax *malaria.

**Conclusion:**

The observed difference in polyclonal antibody production seems related to intrinsic activation states of infected individuals, rather than to parasite-antigen specific immune responses. However, it appears influenced by preceding stimuli. This supports the idea that acquired clinical immunity may not exclusively depend on antigen-specific responses, but also on the individual polyclonal reaction.

## Background

Malaria remains an important health problem in sub-Saharan Africa and in some parts of Asia and South America. Resistance to therapeutic drugs and to insecticides, as well as social and environmental changes, are important factors in this situation. Increasing importance is currently given to antibodies in protection against human malaria, especially directed at erythrocytic stages of *Plasmodium falciparum *[1]. However, this protection is relatively unstable, and the precise role of specificities remains unclear regarding the antigenic variability of parasite proteins. Although parasite-specific antibodies clearly contribute to protection, it is not evident to which extent protective antibodies in general originate from specific immune responses to parasite antigens. They may also include components of innate immunity and be in part derived from natural antibody repertoires of the host. The remarkable protective properties of natural antibodies have previously been demonstrated in viral and bacterial [2-4] as well as in *Leishmania *[5] infection, and it appears conceivable that analogous properties exist toward *Plasmodium *parasites, either naturally or selected during the long co-evolution of the human species with these parasites. An example may be autoantibodies to 'band 3', a host-encoded target on cell membranes, involved in protection against malaria [6,7] as well as physiologic cellular life span regulation [8].

Natural antibodies, immunoglobulins circulating in the absence of particular immunogenic stimuli, emerge from continuous autonomous activity of the immune system which appears largely independent of any external priming, as demonstrated e. g. in germ-free and antigen-free mice [9,10]. They are often multireactive, and a large proportion interacts with endogenous targets and may play roles in internal homeostasis [11]. Reactivity patterns of IgM and IgG natural antibodies to autologous tissue proteins appear established early in life and remain remarkably stable throughout healthy living [12], but are capable of characteristic changes in autoimmune [13,14] and other [15,16] human diseases. Thus, such patterns are likely to reflect stabilized states of physiologic activation, shaped by polymorphic genes relevant for the immune system [17] which can be selected in evolution.

The aim of this study was to investigate whether likely natural antibody reactivity patterns measured toward targets not related to parasites, but rather derived from autologous tissue or intestinal flora, could differentiate between asymptomatic and symptomatic forms of malarial infection. Asymptomatic infection is frequent in the Brazilian Amazon [18], even without very long exposure times, although malaria is only hypo- to mesoendemic and transmission is unstable with seasonal fluctuations [19]. Nevertheless, the situation appears analogous to hyperendemic regions, where parasite loads are known to gradually diminish with exposure time, resulting in a state of premunition, in which the still chronically infected subjects nevertheless remain asymptomatic for long periods [20,21]. Asymptomatic infection can also be maintained after clinical cure by antimalarial drugs [22], showing that protection from disease can be very distinct from parasite clearance, which may paradoxically even enhance the risk of clinical relapses [23].

Asymptomatic and symptomatic subjects who are studied here were occasionally detected in a survey study of endemic Brazilian populations. They mainly consisted of migrants who lived in endemic areas for individually different time periods, and may have experienced sequential infections by *P. falciparum *or *Plasmodium vivax*, with clinical symptoms of variable degrees of intensity but low reported mortality [24]. Results showed that asymptomatic and symptomatic states could be remarkably well distinguished by multi-specific antibody reactivity, particularly when exposure times were limited, and that the distinction consisted more in a difference in nonspecific polyclonal activation than in a specific immunization effect.

## Materials and Methods

### Study areas and subjects

78 sera analysed here originated from subjects exposed to transmission in the Brazilian Amazon endemic area, who had variable numbers of reported previous episodes of *P. vivax *or *P. falciparum *clinical malaria. The malaria-exposed subjects comprised three distinct groups [see Additional file 1]. The principal study group was derived from a screening of 531 miners living in gold-mining areas in the municipality of Apiacás (AP), Mato Grosso, among whom 99 had been found parasitaemic, presenting with positive Giemsa-stained thick-blood smears [25]. Out of these 99 subjects, only 46 had shown classical malaria symptoms within 72 hours after parasite detection, while the other 53 had remained asymptomatic. Symptoms were mainly headache, anorexia and fever. Included in the present study are 48 of these miners, 24 symptomatic and 24 asymptomatic, who had lived for up to 17 years in Apiacás. There was no significant difference between symptomatic and asymptomatic individuals in their time of residence. According to anamnestic reports, numbers of previous malaria episodes were highly variable, but neither significantly different between the groups. The time elapsed since the respective last episode, however, was significantly longer among the asymptomatics (see Table 1). Within these parasitaemic subgroups, subjects were further distinguished whether or not they were positive for hepatitis B surface antigen (HbSAg) and therefore hepatitis B virus (HBV) carriers. Two further groups included exposed, but aparasitaemic control subjects. The first group (20 subjects) had resided for 10 or more years in Terra Nova Norte (TNN), a small rural community within the endemic region, continuously exposed to malaria. These parasitologically negative, convalescent individuals had been treated for malaria only until two months before the blood collection (Aparasitaemic/Previous malaria). The second group (10 subjects) also lived in TNN, but had no record of previous malarial episodes (Aparasitaemic/No previous malaria). All subjects responded to a questionnaire including information on past malaria and previous treatments. Consent to draw blood was obtained from each individual according to the Fundação Oswaldo Cruz Ethics Committee (MH, November 26^th^, 1994) and to the Universidade Federal de Minas Gerais Ethics Committee (April 15^th^, 1998). Venous blood samples (20 ml per subject) were drawn in Vacutainer™ (Becton Dickinson, Oxnard, CA) heparinized tubes. Giemsa-stained thick-blood smears were examined at this point for blood parasitaemia. Finally, a third control group consisted of 10 healthy adult volunteers from Belo Horizonte (Minas Gerais) who had never been exposed to malaria transmission or visited endemic regions (Aparasitaemic/Not exposed). IgG and IgM to human brain proteins were assessed in the subjects described above. When measuring IgG reactivity to *E. coli *proteins, two of the Terra Nova and seven of the Apiacas subjects were not included, but replaced by one Terra Nova and eight Apiacas subjects not assayed for reactivity to brain proteins.

**Table 1:** Composition of the sample studied

Group	Origin	Nb	Age^1^	Residence^1^	Previous malaria^2^	Time elapsed since last episode^1^
Aparasitaemic Not Exposed	B. Horizonte	10	27–55 [31]		0	
Aparasitaemic – No Previous Malaria	Terra Nova	10	16–39 [27]		0	
Aparasitaemic – Previous Malaria	Terra Nova	20	14–35 [27.5]^4^		1–11 [3]^3^	
Parasitaemic – HBV-negative Asymptomatic	Apiacas	19	20–47 [30]	2–13 [8]^4^	2–70 [15]	0–13 [2 years]^3^
Parasitaemic – HBV-negative Symptomatic	Apiacas	13	6–66 [27]	2–17 [7]^3^	2–50 [15]	0–17 [1 month]
Parasitaemic – HBV-positive Asymptomatic	Apiacas	5	20–50 [27]	2–11 [6]	7–50 [15]	0–4 [1 year]
Parasitaemic – HBV-positive Symptomatic	Apiacas	11	24–46 [30]	5–11 [10]^4^	5–50 [35]	0–2 [2 months]

^1 ^In years; range [median]

^2 ^Number of malaria episodes anamnestically reported; range [median]

^3 ^Unknown for one subject

^4 ^Unknown for two subjects

### Parasitaemia and anti-P. falciparum reactivities

The parasite density was quantified after examination of 200 microscopic fields at 1.000× magnification under oil-immersion. All slides were examined by three well-trained microscopists. Blood parasitaemia was expressed as the number of parasites per 200 leukocytes. Parasitaemia and reactivity to *P. falciparum *and MSP1-19 in these populations have been analysed and described previously [25].

### Immunoblot Assay

The assay was done as described [26]. Briefly, protein extracts from human brain and cultured *E. coli *were run in a discontinuous SDS-PAGE 10% gel (Mighty Small electrophoresis apparatus, Hoefer Scientific Instruments, San Francisco, CA). After eletrophoresis, proteins were transferred onto a nitrocellulose membrane (Schleicher & Schuell, Germany) in a semi-dry system for one hour at 0.8 mA/cm^2^. After overnight blocking of free binding sites in PBS-Tween 0.2%, membranes were incubated for four hours with sera, diluted 1/20, in incubation units fixing membranes in cassettes with 28 independent channels (Miniblot System C-Shell, Immunetics Inc., Cambridge, MA). Whole membranes were then washed and incubated for 90 minutes at room temperature with secondary anti-human IgM or IgG conjugated with alkaline phosphatase (Southern Biotech, Birmingham, AL). Reactivies were revealed with NBT/BCIP (nitroblue-tetrazolium/bromo-chloro-indolyl-phosphate) substrate (Promega, Madison, WI) for three to five minutes, and membranes scanned in 8-bit grayscale and with 600 dpi resolution in a domestic scanner (Apple Color OneScanner). Thereafter, membranes were stained overnight with colloidal gold (Biorad, Hercules, CA) to reveal total protein and again scanned as described.

### Data processing and statistical analysis

The method of data processing has been described [26]. Briefly, reactivity profiles from the scanned immunoblot image were adjusted for migration irregularities during electrophoresis, using the scan of the same membrane stained for total protein. Special procedures programmed with the software IgorPro (Wavemetrics, Lake Oswego, OR) on a Macintosh computer (Apple computers, Cupertino, CA) were applied in order to represent each serum sample as a profile on a standardized migration scale, with the optical density (OD) as a function of migration distance. After this rescaling, sections were defined by intervals on the standardized migration scale around reactivity peaks, and, after baseline subtraction, reactivity for each section and serum was quantified as the average OD within a respective section. In this way, each serum can be represented by a vector with a dimension equal to the number of sections, containing the respective reactivity quantitation. In order to make these data commensurate across membranes, they were further normalized by the membrane-wise average reactivity of a unique standard (pool of human IgM or IgG), assayed twice on each membrane.

These vectors were then analysed by Principal Component Analysis (PCA). PCA is a classic method of multivariate analysis designed to describe multidimensional data with high dimensionality through projection onto characteristic subspaces with lower dimensionality. Principal components are defined in a mathematically strict manner as orthogonal axes fitting maximal information in terms of total variance with decreasing proportion and uncorrelated among each other. This includes no information on experimental groups or other particularities, but provides a completely neutral and unbiased description of the data set as such.

The number of bands detected on a respective extract was quantified as the number of sections showing reactivity values two-fold above the average reactivity of the standard used for adjustment. Total reactivity for a given extract was assessed as the average OD over the entire migration scale, from the first to the last section. Only distribution-independent statistics were used: Mann-Whitney rank sum test and Spearman rank correlation. P-values below 0.05 were considered significant.

## Results

### IgG reactivity to human brain proteins

**Figure 1:**
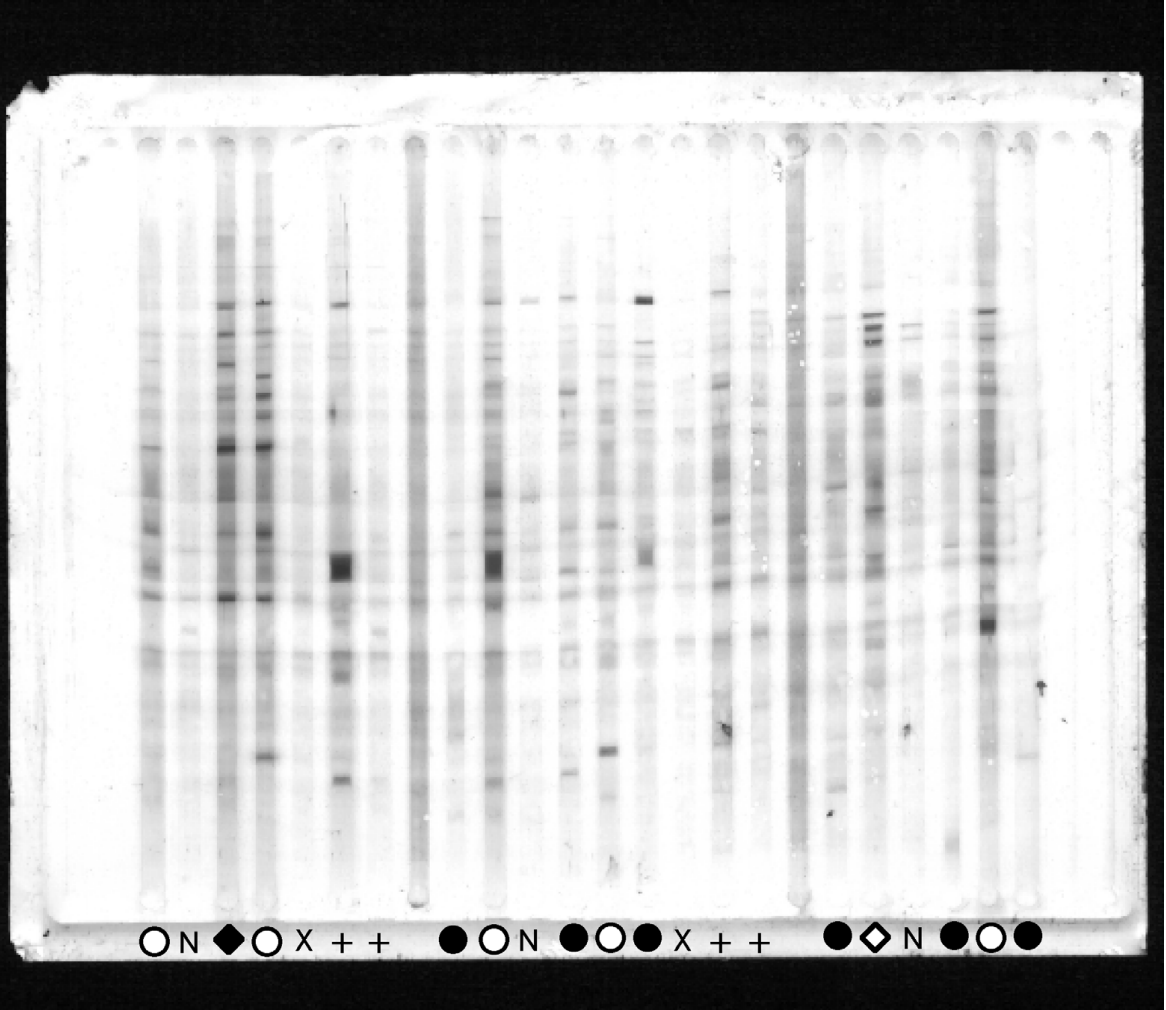
IgG reactivity patterns to brain proteins on one of four membranes. Open circles indicate asymptomatic malaria, closed circles symptomatic malaria in HBV-free parasite carriers, open and closed rhombi analogously in HBV-coinfected subjects. N indicates unexposed (Belo Horizonte), X and + exposed aparasitemic subjects from Terra Nova without and with previous malaria, respectively. Unmarked intermediate lanes contain the standard used for adjustment.

**Figure 2:**
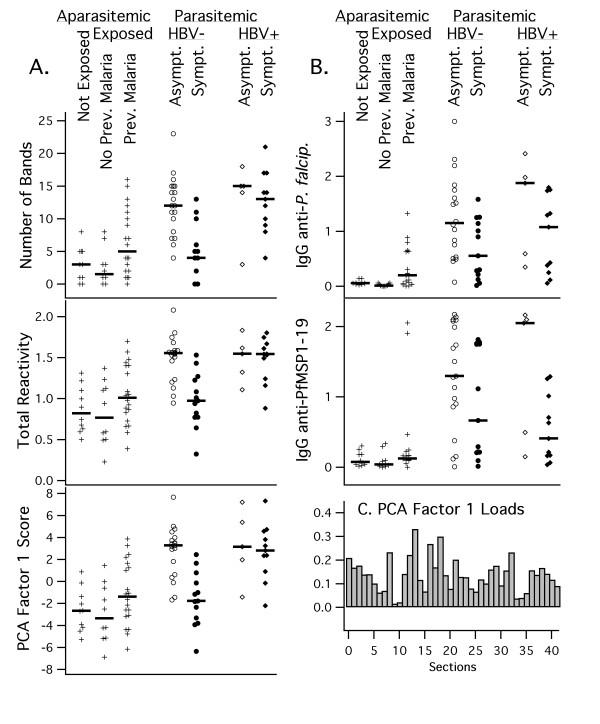
Comparison of properties of anti-brain IgG immunoblot reactivity andantiplasmodial IgG. A. Immunoblot of IgG to human brain proteins: number of bands, total reactivity and PCA factor-1 scores for IgG reactivities for each patient group. B. Antiplasmodial IgG: equivalent displays of data from the same patients for IgG reactivity to anti-*P. falciparum *and anti-PfMSP1-19. In both panels, medians for each group are indicated by vertical bars. C. factor loads for PCA factor-1 shown in panel A.

As an example, Fig. 1 shows one representative out of four total immunoblot membranes on which IgG reactivities to brain proteins were assayed. Most of the reactivity bands appear in samples derived from asymptomatic parasitaemic subjects, with the exception of one symptomatic coinfected with HBV. In Fig. 2, three different (however, correlated) ways to evaluate the reactivity quantitatively are shown : (1) by the number of detected bands; (2) by the summation of total reactivity in terms of averaged optical density over the whole migration scale; (3) by the score of the first principal component calculated from standardized optical densities of all reactivity bands. By any of these criteria, asymptomatic parasite carriers from Apiacas showed more reactivity than parasite-free subjects in all groups with high significance (p < 0.00001). Parasite carriers with symptoms, however, also had less reactivity and, provided that they were not coinfected with HBV, did not significantly differ from parasite-free subjects. Among symptomatics, only HBV-positives showed reactivity levels comparable to the asymptomatics. In the absence of HBV-coinfection, asymptomatic and symptomatic subjects differed with high significance in terms of all three reactivity measures (p = 0.0003, 0.0002 and 0.0001, respectively). Even disregarding the presence of HBV coinfection, this difference was still significant (p = 0.017, 0.008 and 0.005, respectively). Assays of specific anti-parasite reactivity in the same subjects (reported in [25]) are shown in Fig. 2 for comparison. Although showing the same tendency, these specific assays were less discriminatory, considering either only HBV-negative (IgG anti-*P. falciparum*: p = 0.034; IgG anti-MSP1-19: nonsignificant) or all parasitaemic individuals (p = 0.017 and p = 0.014, respectively).

The first principal component (PCA factor 1) is, by definition, the linear combination of single reactivity measurements which represents a maximum of information about a multivariate dataset in terms of total variance, here 34%. Since this is the most systematic quantitative representation, the analysis will be continued based on principal components. In PCA, factor loads, i. e., coefficients indicating the relative contribution of measured parameters to the respective factor score, can be interpreted according to whether this factor represents a general level difference or a pattern-related property. Here, the factor-1 score contained only positive loads and, thus, represents a modified measure of total reactivity. Factor-1 scores indeed showed similar groupwise distributions as band number or total reactivity and were most discriminating. Among the first few principal components, factor 1 was the only one with interpretable properties.

**Figure 3:**
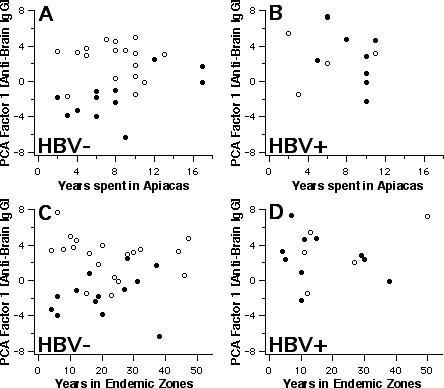
Polyclonal reactivity and exposure. Scores of PCA factor-1, derived from IgG reactivity to brain proteins, in relation to the time spent in Apiacas and in malaria-endemic zones, for HBV-negative (left) and HBV-positive subjects (right). Open symbols indicate asymptomatic, closed symbols symptomatic subjects.

**Figure 4:**
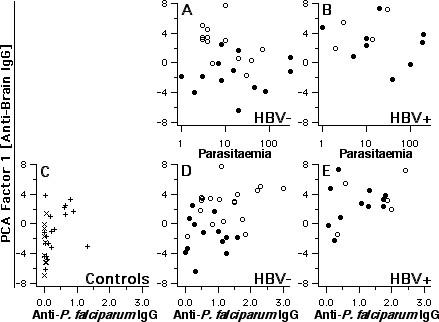
Polyclonal reactivity, parasitemia and anti-PF antibodies. Relations of anti-brain IgG-derived PCA factor-1 scores are shown to parasitemia levels and anti-*P. falciparum *reactivity (ELISA absorbance, according to ref. 27). X and + indicate exposed aparasitemic subjects without and with previous malaria, respectively. The parameters were not always available from all subjects. Open symbols represent asymptomatic and closed symbols symptomatic subjects, all HBV-free.

Further properties of factor-1 scores derived from IgG anti-brain reactivities are shown in Fig. 3, 4, 5. Parasitaemic subjects had provided information on the time they had spent in Apiacas and in malaria-endemic areas in general. When plotted against these exposure times (Fig. 3), factor-1 scores discriminated best between asymptomatic and symptomatic subjects without HBV coinfection who had been exposed for relatively shorter periods. Hence, HBV-free subjects living less than 10 years in Apiacas or less than 20 years in malaria-endemic areas were almost completely separated according to their clinical status by factor-1 scores, but not those with long-term exposure. HBV-coinfected subjects, however, all displayed enhanced scores with no evident relationship to malaria exposure. For instance, both exposure parameters did not differ significamtly between symptomatic and asymptomatic subjects, regardless whether HBV infection is taken into account or not.

**Figure 5:**
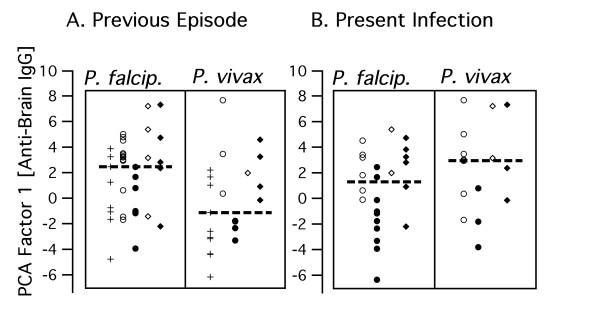
Polyclonal IgG to brain proteins and *Plasmodium *species in previous and present infections. Association of anti-brain IgG derived PCA factor-1 scores and Plasmodium species detected in the previous clinical Malaria episode (A), or presently (B). Medians calculated for all indicated subjects are shown by vertical bars. Subjects in which both species were detected at the same time were not considered. Crosses indicate exposed aparasitemic subjects with previous malaria, circles HBV-, and rhombi HBV+ parasite carriers. Open symbols represent asymptomatic and closed symbols symptomatic subjects.

Fig. 4 shows the relationship between factor-1 scores and (a) individual parasitaemia and (b) anti-parasite IgG. Within HBV-negative asymptomatic subjects, scores were highest when parasitaemia levels were relatively low, although this association was quantitatively insignificant. A significant correlation was found between factor-1 scores and IgG reactivity to *P. falciparum *(Spearman rank correlation: +0.57 [p < 0.00001] including parasitaemics and exposed aparasitaemics; +0.37 [p = 0.011] within parasitaemics only), or to PfMSP1-19 (Spearman R, considering parasitaemics and exposed aparasitaemics: +0.42 [p = 0.0002]; not shown). Nevertheless, as can be seen in Fig. 4 for IgG anti-*P. falciparum*, these correlations are mainly due to the fact that all parameters were relatively enhanced in asymptomatic subjects. Considering asymptomatic and symptomatic individuals separately, correlations are no longer evident. Namely, some HBV-free symptomatics had elevated IgG anti-*P. falciparum*, but without a parallel increase in factor-1 scores.

Medical records of previous episodes of clinical malaria were available for both parasitaemic and exposed aparasitaemic subjects. Considering subjects of all groups together, factor-1 scores of individuals with evidence for *P. falciparum *infection in their last clinically diagnosed episode were higher than for those who had had *P. vivax *malaria (Mann-Whitney test: p < 0.01). Qualitatively, this appeared to be the case for parasite carriers as for aparasitaemic subjects with reported previous malaria (Fig. 5), although a valid statistical evaluation taking subject groups into account is impossible due to the small sample size. Nevertheless, it is interesting that the *Plasmodium *species presently detected in the parasitaemic subjects, often not identical with the one ascribed to the previous malaria period, was not associated with a similar difference in respect to factor-1 scores.

### IgG reactivity to E. coli extract

**Figure 6:**
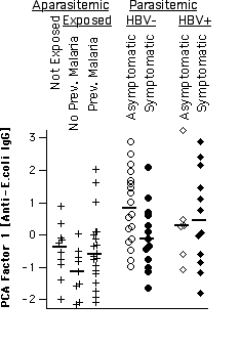
IgG reactivity to *E. coli *proteins. Distributions of PCA factor-1 scores derived from IgG reactivities to *E. coli *proteins per group. Group medians are indicated by vertical bars.

**Figure 7:**
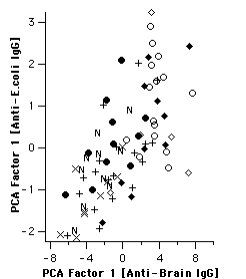
IgG reactivity to different antigenic sources is highly correlated. IgG anti-brain and anti-*E. coli *derived PCA factor-1 scores, respectively, are displayed in two dimensions. N indicates non-exposed, X and + exposed aparasitemic subjects without and with previous malaria, respectively (B,C). Circles indicate HBV-, rhombi HBV+ parasitemics, open symbols asymptomatic and closed symbols symptomatic subjects.

**Figure 8:**
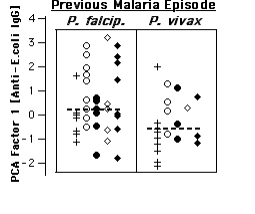
Polyclonal IgG to *E. coli *proteins and *Plasmodium *species in previous infection. IgG anti-*E. coli *derived PCA factor-1 scores are displayed against the parasite species detected in the previous malaria episode, in analogy to Fig. 5. Medians indicated by vertical bars include all subjects in the figure. Crosses represent exposed aparasitemic subjects with previous malaria, circles HBV-, and rhombi HBV+ parasite carriers. Open symbols represent asymptomatic and closed symbols symptomatic subjects.

Patterns of IgG reactivity were also assayed toward an extract of *E. coli *whole cultures, which provided an independent and completely different set of target antigens, and, consequently, a different set of reactivity bands. Surprisingly, multivariate analysis of these bands yielded a result very similar to that described above for IgG reactivity to human brain proteins (Fig. 6). Although less significantly, the *E. coli*-derived score shared several properties described above. Analogously, asymptomatic parasitaemics had higher scores for reactivity to *E. coli *than symptomatics in the absence (p = 0.041), but not in the presence of HBV coinfection. Also similarly to IgG anti-brain, this significance increased when only subjects living less than 10 years in Apiacas were considered (p = 0.002, not shown). As can be seen in Fig. 7, factor-1 scores calculated from anti-*E. coli *IgG reactivities indeed correlated highly with anti-brain-IgG-derived factor-1 scores (Spearman rank correlation: +0.70; p < 1E-9). Finally, IgG anti-*E. coli*-derived factor-1 scores were higher in subjects reporting previous infection with *P. falciparum*, compared to *P. vivax *infection (p < 0.05; Fig. 8).

**Figure 9:**
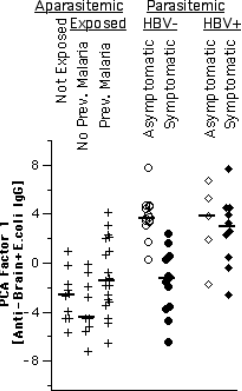
IgG reactivity to *E. coli *proteins. Distributions per group of PCA factor-1 scores derived from IgG reactivities to human brain and *E. coli *proteins joined together. Group medians are indicated by vertical bars.

When principal components were calculated from IgG reactivities to both brain and *E. coli *proteins together, the properties described for both respective factor-1 scores fell together in the resulting first principal component (Fig. 9), indicating again that they were highly coincident. The difference between HBV-free asymptomatic and symptomatic parasitaemic subjects reached a higher significance level (p = 0.00005) than for factor-1 scores derived from either extract alone.

### IgM reactivity to human brain proteins

IgM reactivities to brain proteins were measured using the same extract as described for IgG (data not shown). Generally, a smaller number of bands was detected. The IgM-derived factor-1 included only positive loads like the IgG-derived one. The scores of both were correlated (Spearman rank correlation: +0.55; p < 1E-6), and, as for IgG, IgM-derived factor-1 scores were higher in asymptomatic than in symptomatic parasitaemic individuals free of HBV. However, IgM-derived factor-1 did not significantly discriminate between them, except when only individuals living less than 10 years in Apiacas were considered (p = 0.013). No significant difference was found either in respect to previously or presently detected *Plasmodium *species. In contrast to IgG anti-brain or anti-*E. coli*, within parasitaemics, a remarkable positive correlation existed between IgM-derived factor-1 scores and individual age (Spearman rank correlation: +0.41; p = 0.005), and also with times spent in Apiacas (+0.34; p = 0.03), but not within aparasitaemic subjects. As for IgG anti-brain, there was no significant correlation with blood parasitaemia levels, but with specific IgG anti-*P. falciparum *(+0.55; p < 1E-5).

### Joint analysis of IgG and IgM reactive to brain proteins reveals distinct components

**Figure 10:**
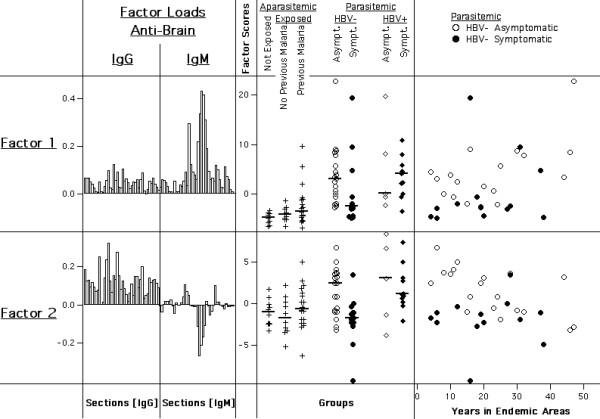
Principal components derived from IgG and IgM reactivities to human brain taken together. A. Coefficients of factor 1 and 2, representing weight and direction of section reactivities contributing to the respective factor scores. B. Distributions of factor 1 and 2 scores according to subject groups. Vertical bars represent medians within each group. C. Distribution of factor 1 and 2 scores in respect to Malaria exposure (HBV-free parasite carriers only). Different subject groups are represented by the indicated symbols.

Finally, principal components were calculated for IgG and IgM reactivities to human brain proteins together (Fig. 10). Surprisingly, the properties of the respective first factors calculated for each isotype alone did not coincide in a single principal component, as it had occurred in the co-calculation of IgG anti-brain and anti-*E. coli *reactivities. Instead, the first two principal components (factor-1 and factor-2) of the co-calculation both significantly distinguished between HBV-free asymptomatic and symptomatic parasite carriers (Factor 1: p = 0.013; Factor 2: p = 0.004), and, as above, not between those infected with HBV. Scores of both factors were enhanced in asymptomatic subjects. Since principal components in the same set are by definition uncorrelated, these two factors appear to represent separate effects associated with clinical states.

Factor-1, characterized by positive loads as the above described, was however positively influenced primarily by IgM reactivity, and properties of factor-1 scores were similar to those of the IgM-derived factor-1. Within parasitaemic subjects, scores increased with age (Spearman R: +0.39; p = 0.007), with exposure times in Apiacas and in endemic areas (+0.29 and +0.26, but not significant), and were correlated with anti-*P. falciparum *IgG (+0.32; p < 0.05). However, like IgG-derived factor 1, scores were also elevated in respect to *P. falciparum *but not *P. vivax *pre-exposure (p = 0.01; not shown).

In contrast, factor-2 scores were positively influenced mainly by IgG reactivity, while a number of IgM bands had a negative impact. Thus, factor 2 can be said to represent a certain IgG/IgM relationship. Remarkably, among HBV-free parasite carriers, the highest scores were characteristically found in asymptomatics who had only been exposed to malaria in endemic areas for a limited time. Factor-2 scores showed no significant association with IgG anti-*P. falciparum *or with pre-exposure to *Plasmodium *species. Instead, scores were slightly higher in subjects presently infected with *P. vivax*, but not *P. falciparum *(p = 0.04; not shown). Neither factor correlated significantly with parasitaemia.

### Association with reactivity to MSP1-19

Global IgG reactivity and subtype-specific reactivity have been previously assayed including some subjects examined here [25]. Among parasitaemic subjects, IgG1, IgG2, IgG3 and IgG4 anti-MSP1-19 all correlated significantly with IgM-anti-brain-derived as well as with IgG/IgM-anti-brain-derived factor-1, in particular IgG3 (Spearman R: +0.68 [p < 0.001] and +0.62 [p < 0.01], respectively). These values, however, did not corrrelate significantly with any IgG-derived factor, nor with IgG/IgM-anti-brain-derived factor-2. Only IgG anti-MSP1-19, measured in a larger number of subjects, correlated positively with IgG-anti-brain and IgG-anti-brain/*E. coli*-derived factor-1 (not shown).

## Discussion

Autoantibodies detected in the context of malaria infection are an old observation, reviewed in [27,28]. Typically, they were found elevated in long-term exposed subjects and in acute symptomatic infection, while asymptomatic infection has rarely been studied. However, phospholipid-related specificities were found more elevated in asymptomatics than in patients with malaria symptoms [29,30]. All these previous studies focused on antibody specificities otherwise known from autoimmune disease conditions, particularly systemic lupus erythematosus. The findings were often interpreted as results of specific crossreactivity or auto-immunization, and analogies were regularly made to 'autoimmunity'. This appears confusing, since malaria infection and pathological autoimmunity have little obviously in common and, in fact, the respective pathologies appear to mutually inhibit rather than to facilitate each other [27].

The simultaneous standardized detection of multiple immunoglobulin reactivities on immunoblots, as utilized in the present study, is most likely to represent natural antibody repertoires and, therefore, a way to approach the problem of repertoire description in an unbiased way, avoiding a potentially misleading disease- or otherwise antigen- or specificity-oriented vision. Extracts of human tissue (brain) or whole bacterial cultures were used as ligands for antibodies with the aim of relating eventual changes in binding patterns to clinical status, and comparing whole reactivity patterns by multivariate analysis. The same semiquantitative immunoblot technique has previously demonstrated characteristics of natural IgM and IgG reactivity patterns [17,31,32], among which stability [12,33,34] and independence of physiologic antigenic exposures [35] are most remarkable. IgG patterns, however, change with age [36], during human [13,14] and experimental [37] autoimmune diseases and in murine parasite infection [38].

The present results show that reactivity patterns to human brain and *E. coli *proteins, unrelated to parasites, could differentiate strikingly between asymptomatic and mild symptomatic malaria infection. Asymptomatic subjects generally showed elevated reactivity, which was concordant but far more characteristic when compared with anti-plasmodial IgG measures in the same sample. Thus, *Plasmodium *infection is able to alter reactivity patterns to target proteins without relation to the parasite particularly in asymptomatic subjects. Only co-infection with HBV, known to be frequent in our study population [39], heightened reactivity regardless of malaria symptoms and obscured this discrimination when disregarded. This effect is most probably unrelated to malaria, since chronic HBV infection itself is known for its association with a variety of autoantibodies [40]. Remarkably similar results were obtained by analyses of IgG immunoblot reactivity against extracts of human brain and of *E. coli*. In both cases, the same major properties were represented by a single respective principal component, characterized by positive loads. Both were highly correlated and when reactivity to both extracts was analysed together, they coincided in a single PCA factor with maximal discriminatory power, allowing to separate 13/15 (87%) of the HBV-free asymptomatics from symptomatic patients. Thus, IgG reactivity patterns to brain and to *E. coli *proteins behaved somewhat like fragments of a hologram which, added together, show the same image with increased resolution.

Other than previous data on malaria-associated autoantibodies, these observations cannot easily be attributed to specific immune responses, despite a positive correlation with anti-parasite reactivity. Instead, they probably reflect another phenomenon well known for malaria and other parasite infections, that of polyclonal lymphocyte activation. Polyclonal activation, however, is often discussed as an immune evasion mechanism, beneficial primarily for parasites. Our results show in contrast that the production of polyclonal IgG was associated with protection in asymptomatic parasite carriers, and that polyclonal reactions appeared to have at least short-term protective effects. A reason for this discrepancy may be that earlier studies of polyclonal activation [41] assessed peripheral plaque-forming cells, which may not contribute much to circulating antibody repertoires, and not the recruitment of resident plasma cells with a relevant lifespan from which circulating natural antibodies likely originate. When such natural antibodies as they are addressed by us have pre-existent protective properties, their polyclonal recruitment can well be efficient and protective, as it has been demonstrated for other infections [2,3,5]. This could be due to preceding evolutionary selection. Our observation of an effect of the parasite species present in previous clinical episodes further suggests that previous antigenic experience can trigger not only classical recall responses, but also this capacity to react polyclonally. With increasing exposure time, this nonspecific recruitment of natural repertoires could be stepwise complemented by induced parasite-specific antibodies, leading to an increasingly adapted protection as it is observed in long-term exposed subjects.

Remarkably, in our study, asymptomatic and symptomatic infections were indeed best discriminated in subjects with relatively short exposure times. In contrast to previous data on autoantibodies, this indicates that the reactivity shift in asymptomatic subjects described here does not result from long-term adaptation to the parasite, but is representative of an intermediate state of adaptation. This interpretation is most clearly supported by the result of joint analysis of IgM and IgG reactivity to brain proteins, where two distinct principal components appeared. Factor-1, representing a general elevation of all reactivities with dominant IgM impact, shows an evident overall time dependency in parasite carriers and may, thus, reflect long-term adaptation. However, factor-2 discriminated asymptomatic from symptomatic infections best and, most characteristically, those asymptomatic subjects who had been least exposed. Factor-2 represented a relative dominance of IgG reactivity against some IgM. Thus, in order to remain asymptomatic, particularly infected subjects without long-term adaptation to the parasite may require a pattern of natural antibody production dominated by IgG but not IgM.

This is in principal agreement with known protective effects of antibodies. Classically, passive transfer of hyperimmune IgG to patients infected with *P. falciparum *has shown that antibodies play a crucial role in controlling blood stage parasitaemia [42,43]. The gradual acquisition of clinical immunity to malaria after repeated infection is positively correlated with the development of a diverse IgG repertoire, most clearly including IgG reactive to parasitized red blood cells [1]. Such cytophilic antibodies are predominant in clinically immune individuals living in hyperendemic areas [21]. In contrast to IgG, IgM with the same cytophilic properties is associated with rosetting and enhanced pathogenicity [44-47].

The targets of cytophilic antibodies include diverse and highly variable parasite-encoded proteins, but also host proteins such as 'band 3' [6], which is involved in parasite entry into red blood cells [7]. Nevertheless, anti-band 3 antibodies may just exemplify the possible relevance of natural repertoires, since they are originally known as physiologic autoantibodies involved in the general clearance of senescent and damaged (including infected) erythrocytes [8]. Intrinsic tuning of the production rate of such pre-existing antibodies may, therefore, contribute as much to the control of parasitaemia that occurs in clinically immune individuals, as do cytophilic antibodies originating from parasite-specific responses. Other natural antibody-mediated effects could act analogously, and an appropriate individual reshaping of the natural repertoire toward such components could well lead to a capability to regulate parasitaemia, triggered by parasite-mediated polyclonal lymphocyte activation. Such reshaping, associated with a shift in internal self-recognition, could also explain the appearance of lupus-like autoantibodies in chronically exposed subjects [28] without, however, indicating autoimmunity. In general, protective natural repertoire components appear to deserve further investigation, since vaccines and other malaria control strategies could possibly be designed to make use of them.

## Conclusions

This study shows that simultaneous detection of IgM and IgG reactivities to a broad range of targets can reveal remarkable properties which are not easily accessible by the usual specificity-focussed approaches. Thus, clinical states in *Plasmodium *infection appear to depend on internal activation states which can be distinguished by different patterns of polyclonal antibody production. Particularly, asymptomatic but not symptomatic infection of HBV-free subjects without extensive pre-exposure to malaria was characterized by elevated polyclonal IgG reactivity in absolute terms and in relation to IgM, furthermore appearing to be triggered by previous *P. falciparum *but not *P. vivax *infections.

## Authors' contributions

CJFF and EMB did the field work and collected the samples. NMV designed and supervised the study. AC performed preliminary experiments. CF, LFG and ASN did the immunoblots and analysed the results. CF prepared software, performed statistics and wrote the manuscript.
